# Epothilone B Benefits Nigral Dopaminergic Neurons by Attenuating Microglia Activation in the 6-Hydroxydopamine Lesion Mouse Model of Parkinson’s Disease

**DOI:** 10.3389/fncel.2018.00324

**Published:** 2018-09-28

**Authors:** Zhongyuan Yu, Ling Yang, Yang Yang, Siyu Chen, Dayu Sun, Haiwei Xu, Xiaotang Fan

**Affiliations:** ^1^Department of Developmental Neuropsychology, School of Psychology, Third Military Medical University, Army Medical University, Chongqing, China; ^2^Battalion 3 of Cadet Brigade, Third Military Medical University, Army Medical University, Chongqing, China; ^3^Department of Neurosurgery, Southwest Hospital, Third Military Medical University, Army Medical University, Chongqing, China; ^4^Southwest Eye Hospital, Southwest Hospital, Third Military Medical University, Army Medical University, Chongqing, China

**Keywords:** EpoB, Parkinson’s disease, substantia nigra, microglia, inflammatory factors

## Abstract

Parkinson’s disease (PD) is characterized by loss of dopamine (DA) neurons in the substantia nigra pars compacta (SNc) and a subsequent reduction in striatal DA levels. Recent studies have shown that systemic administration of subtoxic doses of epothilone B (EpoB), a microtubule stabilizing agent, enhances axonal regeneration. However, the underlying alterations in cellular mechanisms remain undetermined. In the present study, we investigated the neuroprotective effects of EpoB on DA neurons in mouse model of PD induced by 6-hydroxyDA (6-OHDA) and *in vitro*. The results indicated that EpoB improved behavioral deficits, protected the nigrostriatal dopaminergic projections and restored DA level in the striatum of mice exposed to 6-OHDA. Meanwhile, EpoB attenuated microglia activation in the SNc of PD mice. Furthermore, EpoB treatment ameliorated 6-OHDA induced cytotoxicity to MN9D dopaminergic cells in a co-culture transwell system of BV2/MN9D cells, and redistributed the cytoskeleton of microglial BV2 and caused the morphological transition, inhibited the polarization to the M1 phenotype by suppressing expression of pro-inflammatory factors including interleukin (IL)-1β, IL-6 and tumor necrosis factor (TNF)-α. Overall, our study suggested that EpoB treatment protects nigral DA neurons and projections through limiting the cytotoxicity of activated microglia in 6-OHDA lesioned mice.

## Introduction

Parkinson’s disease (PD) is one of the most common and debilitating age-associated human neurodegenerative disorders, characterized by a variety of symptoms such as resting tremor, bradykinesia, rigidity and postural instability (Mullin and Schapira, 2015; Kabra et al., 2018). The pathogenesis of PD is a progressive loss of dopamine (DA) neurons in the substantia nigra pars compacta (SNc) and a subsequent reduction in striatal DA levels (Kulisevsky et al., 2013). The present clinical intervention for PD aims principally to ameliorate the clinical manifestations of the disorder (Jeon et al., 2010; Kulisevsky et al., 2013), there is no effective cure that can stop or slow the degeneration of DA neurons.

Recent studies in animals and humans have demonstrated that axon degeneration might be an early pathological event that indicates damaged DA neurons and might be a key cause of PD (Branchi et al., 2010; Guo et al., 2015). This seems to infer that preventing axon degeneration at the onset of early pathological events could avoid later degenerative consequences in the DA neurons, which might be another therapeutic strategy for treating PD.

Microtubules are essential for a wide range of dynamic cellular processes in the nervous system and also the main component of axons in DA neurons (Cartelli et al., 2013). It has been suggested that strategies of microtubule stabilization would prevent the breakdown or degeneration of neurites after injury or in neurodegenerative diseases (Lee et al., 2006). Studies in the PD model induced by 1-methyl-4-phenyl-1,2,3,6-tetrahydropyridine (MPTP) or 6-hydroxyDA (6-OHDA) have shown that alteration of microtubules is an early event specifically associated with DA neuron degeneration (Cartelli et al., 2013). Pharmacological stabilization of microtubules might be a viable strategy for the management of parkinsonism.

Epothilones are a class of FDA-approved blood-brain-barrier (BBB) permeable antineoplastic agents known to induce α-tubulin polymerization and enhance microtubule stability (Ballatore et al., 2012). Epothilone B (EpoB) has been shown to induce cell apoptosis of the human ovarian cancer cells OV-90 and ABT-737 through the Apo-2L/TRAIL and PI3K/AKT/mTOR pathways, respectively (Rogalska and Marczak, 2015; Li Y. L. et al., 2016). It has been found that systemic administration of subtoxic concentrations of EpoB enhanced axonal regeneration and attenuated fibrotic scarring after spinal cord injury neither through inhibiting proliferation nor by increasing apoptosis of fibroblasts (Ruschel et al., 2015). It is noteworthy that intraperitoneal administration of low doses of epothilone suppressed axonal microtubule loss and alleviated cognitive defects in a mouse model of tauopathy (Ballatore et al., 2012), and systemic injection of epothilone D was neuroprotective in an MPTP-induced mouse model of parkinsonism (Cartelli et al., 2013). Recently, we demonstrated that EpoB alleviated injury of the nigrostriatal pathway after intracerebral hemorrhage and improved motor function in a mouse model (Yang et al., 2018a), confirming the neuroprotective effects of EpoB.

Although the etiology of PD has not been clarified completely, post-mortem examination, retrospective studies and genetics have implicated a key role of neuro-inflammation in the pathological progress of PD (Kannarkat et al., 2013; Kulisevsky et al., 2013; Gupta et al., 2018). As the main immune cells in the central nervous system (CNS), microglia are activated and produce a large amount of pro-inflammatory cytokines including interleukin (IL)-1β, IL-6 and tumor necrosis factor (TNF)-α, which impact neuronal function and increase cell death in PD (Kannarkat et al., 2013; Moehle and West, 2015). This suggests that microglia can be a therapeutic target for PD since they can be easily modulated by a series of drugs and compounds such as minocycline, spermidine and TSPO ligand XBD173, among others (Trapani et al., 2013; Liu et al., 2018).

Recently, it has been demonstrated that low doses of the EpoB-derived synthetic compound [(S,E)-2-methyl-1-(2-methylthiazol-4-yl)hexa-1,5-dien-ol] (MMHD) inhibited lipopolysaccharide (LPS)-induced activation of BV2 microglia through suppression of the nuclear factor-κB (NF-κB) signaling pathway (Jeon et al., 2010). Considering its ability to penetrate the BBB and stabilize axonal microtubules, it is worth exploring whether EpoB is capable of protecting DA neurons in PD models. However, knowledge is limited regarding the underlying alterations in cellular mechanisms induced by the administration of EpoB. In the present study, we investigated the effects of EpoB treatment in a mouse model of PD induced by injection of 6-OHDA. We verified EpoB treatment could protect against behavioral impairment elicited by 6-OHDA injury. In addition, we investigated the protective effect of EpoB on nigrostriatal dopaminergic projections and DA restoration in the striatum of PD mice and whether the neuroprotection of EpoB against 6-OHDA is mediated by suppression of microglial activation. Our study confirmed that EpoB could be a promising therapeutic candidate for protection of DA neurons in PD mouse models induced by 6-OHDA.

## Materials and Methods

### Animals

Adult male C57/BL6 mice (8 weeks old, 20–23 g) were provided by the Third Military Medical University and were housed in a temperature-controlled room with a standard 12-h light/12-h dark cycle and *ad libitum* access to food and water. All experimental procedures were approved by Third Military Medical University Ethics Committee and were performed according to the guidelines of laboratory animal care and use. All efforts were made to reduce the number of animals used and to minimize their discomfort.

### Drug Treatment

6-OHDA (Sigma, H4381, St. Louis, MO, USA) was dissolved in 0.9% NaCl/0.02% ascorbate at a concentration of 5 μg/μl. Each mouse was pretreated with an intraperitoneal injection of 20 mg/kg desipramine 30 min prior to the unilateral injection of 6-OHDA into the right striatum (anteroposterior: +0.9 mm; mediolateral: −2.2 mm; dorsoventral: −2.5 mm relative to the bregma) at a rate of 0.5 μl/min for a total dose of 15 μg/3 μl as previously described (Kim et al., 2016). The pretreatment of desipramine blocked 6-OHDA uptake by noradrenergic terminals, thereby prevented the destruction of noradrenergic neurons (Kim et al., 2012; Tanaka et al., 2017). The needle was kept in place for an additional 5 min before being slowly retracted. To investigate the neuroprotective effect of EpoB, mice received intraperitoneal injections of EpoB (1.5 mg/kg dissolved in dimethyl sulfoxide (DMSO) and saline at a ratio of 1:3) or an equal volume of vehicle 2 h after 6-OHDA injection.

### Behavior Tests

**Figure 1:**
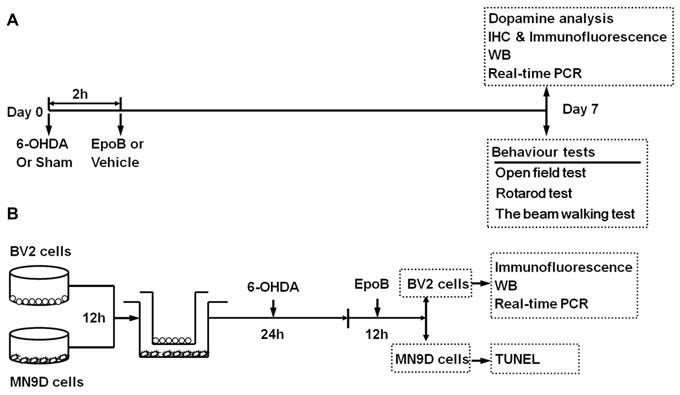
Timeline of study procedures. **(A)** Study of the neuroprotective effect of epothilone B (EpoB) *in vivo*. Mice were injected with 6-hydroxydopamine (6-OHDA) into the right side of the striatum, and 2 h later, the animals were treated with EpoB. Seven days post lesion (Day 7), animals were used for behavioral tests, DA analysis, immunohistochemistry, immunofluorescence, western blot and real-time polymerase chain reaction (RT-PCR). **(B)** *In vitro* experiment, the BV2 microglia and MN9D cells were seeded separately for attachment. After 12 h, BV2 cells and MN9D cells were co-cultured, and 6-OHDA was added to the co-culture system for 24 h. Then, after washed with PBS, the co-culture system was treated with EpoB for 12 h. BV2 cells were collected for immunofluorescence, western blot and RT-PCR, MN9D cells were collected for TUNEL staining.

Seven days after the 6-OHDA lesion operation, the mice were assessed for motor function using an open field test, rotarod test and beam walking test (Buccafusco, 2000; Figure 1A). All behavioral tests were done in a blinded fashion.

### Open Field Test

An open field test was used to measure spontaneous locomotor activity in all mice and conducted as described in our previous study (Xu et al., 2014). The apparatus was made of four transparent Plexiglass walls (40 cm high) and a gray floor of 40 cm × 40 cm. Mice were allowed to freely explore the apparatus for 5 min. The experiments were recorded, and the videos were analyzed using Noldus EthoVision XT software (Noldus Information Technology Co, Ltd.) to determine the total distance traveled.

### Rotarod Test

For motor coordination ability, the mice were evaluated on a rotarod apparatus according to a previous study (Buccafusco, 2000). The animals were acclimated to the apparatus with three prior training sessions. During the test, the acceleration time was set as 35 s and the maximal rotarod rpm was 35. The duration and distance on the rod were recorded (a 300-s maximal time was used for the test).

### The Beam Walking Test

The beam walking test was performed to assess motor coordination and was conducted according to previously described methods (Yang et al., 2018a). A beam of 6 mm width, 1.2 m length and 60 cm height was used in this study. The performance of the mice was videotaped and evaluated by an experimenter who did not know the experimental conditions of individual mice. Hindlimb fault rate was obtained as the average values from three trials.

### Immunohistochemistry and Immunofluorescence

According to our previous method (Zhang et al., 2018), brains of the mice were dissected and fixed in 4% paraformaldehyde (PFA) for 24 h at 4°C and transferred to 30% sucrose for a minimum of 48 h. Serial coronal brain sections (30 μm in thickness) were prepared. The sections were incubated with 0.5% H_2_O_2_ in phosphate buffered saline (PBS) for 30 min to quench endogenous peroxidase and then were incubated with 0.5% Triton X-100 in PBS for 30 min. To block non-specific binding, sections were incubated in 3% bovine serum albumin (BSA) for 1 h at 37°C. Sections were then incubated with the following primary antibodies in 1% BSA (12 h, 4°C): rabbit anti-Iba1 (1:1,000, Wako, CA, USA) or rabbit anti-TH antibody (1:1,000, Millipore, CA, USA). After washing, for TH staining, the sections were incubated with a biotin-conjugated secondary antibody and visualized with bright-field microscopy using a diaminobenzidine substrate kit (Vector Laboratories, Burlingame, CA, USA). For Iba1 staining, the sections were incubated with Cy3-conjugated secondary antibody (1:400, 3 h; Jackson ImmunoResearch, West Grove, PA, USA) and mounted with Vectashield (Vector Laboratories). Nuclei were subsequently stained with 4′,6′-diamidino-2-phenylindole (DAPI, Beyotime, China). The stained cells were observed and photographed with a Zeiss Axivert microscope equipped with a Zeiss AxioCam digital color camera connected to the Zeiss AxioVision 3.0 system (Oberkochen, Germany).

### Immunoreactivity Quantification

TH-positive (TH^+^) cells in the SNc were stereologically estimated in accordance with a previous study (Masoud et al., 2015). Briefly, every fourth section was examined through the entire rostrocaudal extent of the ipsilateral SNc (approximately −2.92 to −3.64 from the bregma; 24 total sections involved). The optical fractionator method was then used to estimate the total number of TH^+^ cells in the SNc of each animal. TH^+^ fibers in the striatum were quantified according to optical density with KS300 software (AxioVision) as previously described (Bao et al., 2017). The relative optical density of each group was obtained by subtracting the background optical density and was normalized to that of the sham group.

The cell count for microglia was performed by analyzing five different SNc sections chosen according to their rostrocaudal coordinates, five mice in each group. Cell density was assessed by counting Iba1^+^ cells from a picture (0.5 mm^2^ frame) taken from the same areas of the same SNc section. Activated Iba1^+^ microglia in the SNc were analyzed based on enlarged and/or amoeboid cell body, thickened processes and an increased staining intensity (Haas et al., 2016). The quantitative data of the total of Iba1^+^ microglia and activated Iba1^+^ microglia were calculated and presented as cells/mm^2^.

### Western Blotting

The western blot analysis was conducted as described previously (Xu et al., 2014). The right substantia nigra (SN) and striatum of the mice were isolated and homogenized in ice-cold RIPA Lysis buffer. After centrifugation of lysates (15,000 g, 5 min at 4°C), the protein concentration was determined via the Bicinchoninic Acid Kit (Beyotime Institute of Biotechnology, Shanghai, China). Protein samples (20 μg per lane) were separated on a 12% SDS-polyacrylamide gel at 80 V for 120 min and then transferred onto polyvinylidene fluoride (PVDF) membranes at 210 mA for 90 min. The membranes were blocked with Tris-buffered saline (TBS) containing 0.1% Tween 20 (TBST) and 5% fat-free milk for 3 h at RT. The membranes were then incubated (overnight at 4°C) with rabbit antibody against TH (1:1,000, Millipore), rabbit antibody against acetylated-α-tubulin (acetyl K40, acetyl-tubulin, 1:1,000), mouse antibody against α-tubulin (1:1,000), and mouse antibody against GAPDH (1:1,000, Cell CWBIO, Beijing, China). Membranes were then incubated for 2 h at RT with a peroxidase-conjugated secondary antibodies (1:1,000). All western blotting data were representative of at least three independent experiments. Specific protein bands on the membranes were visualized by the enhanced chemiluminescence method (Amersham, Piscataway, NJ, USA) according to the manufacturer’s instructions. The relative intensities of TH were normalized to the internal reference protein GAPDH, and the relative intensities of an acetylated-α-tubulin were normalized to the internal reference protein α-tubulin. Three animals per group were used for analysis.

#### Measurement of Monoamine Neurotransmitters and Their Metabolites in the Striatum

The concentrations of monoamine neurotransmitters DA and their metabolites including 3,4-dihydroxyphenylace-tic acid (DOPAC) and homovanilic acid (HVA) in the striatal tissue were determined by high performance liquid chromatography-electrochemical detection (HPLC-ECD), based on the corresponding standard curves. Three mice were selected from each group, and their striata were quickly dissected on ice after euthanasia. Tissue samples were weighed and homogenized in ice-cold 0.1 M perchloric solution (1 mg tissue sample: 10 μl 0.1 M perchloric acid). After centrifugation (12,000 *g*, 30 min), the supernatants were injected into an ECD-HPLC system equipped with a C18 column (Thermo Fisher Scientific, Inc., Waltham, MA, USA) and an ESA detector (ESA, Inc., Chelmsford, MA, USA). The mobile phase consisted of 50 mM sodium citrate phosphate buffer (pH 4.2) supplemented with 8% acetonitrile, 2.4% methanol, 0.25 mM sodium octyl sulfonate and 0.25 mM EDTA. The column temperature was set at 25°C, and the flow rate was maintained at 0.8 mL/min. DA and its metabolites were quantified by peak area comparisons with standards run on the day of analysis. Data were collected and analyzed using CoulArray software.

#### Co-culture of MN9D Cells With Microglia

The immortalized murine microglia cell line BV2 was purchased from the Chinese Academy of Sciences. MN9D cells, a mesencephalon-derived dopaminergic neuronal cell line (Linsenbardt et al., 2012), were a generous gift from the Department of Neurosurgery, Southwest Hospital, Third Military Medical University. Both cell types were maintained in DMEM supplemented with 10% fetal bovine serum, 1% GlutaMax, and 1% penicillin-streptomycin (Gibco, Life Technologies, Grand Island, NY, USA). MN9D cells were seeded onto 6-well plates at a density of 3 × 10^5^ cells/well 12 h for attachment (Li Z. et al., 2016; Wang et al., 2017). BV2 cells were seeded into the transwell inserts (Millipore), which corresponds to a 6-well plate at a seeding density of 2 × 10^5^ cells per well. After attachment, the cells were divided into four groups: (A) Sham+Veh; (B) Sham+EpoB; (C) 6-OHDA+Veh; and (D) 6-OHDA+EpoB. To mimic the activated state of microglia in PD *in vivo* (Keren-Shaul et al., 2017; Krasemann et al., 2017), mixed culture of MN9D cells and BV2 cells was then treated with 50 μM 6-OHDA for 24 h (Xi et al., 2017; Zhang et al., 2017). After this stimulation, cells were washed twice with PBS, and then treated with EpoB (5 nmol/L) for another 12 h (Yang et al., 2018a). BV2 cells were collected for immunofluorescence detection, western blot analysis and real-time polymerase chain reaction (RT-PCR) test, and MN9D cells were collected for apoptosis analysis (Figure 1B).

#### TUNEL Staining

To analyze the apoptosis of MN9D cells, TUNEL staining was performed using the *in situ* Cell Death Detection Kit (Roche, Indianapolis, IN, USA) according to the manufacturer’s instructions. In brief, cells were fixed with 4% PFA, permeabilized with 0.1% Triton X-100 and incubated with a mixture of enzyme solution (TdT) and Label Solution (fluorescein-dUTP; 1:9) for 1 h at 37°C. After washing three times with PBS, the nuclei of the cells were stained with DAPI (Beyotime, China). After immunostaining, the cells were observed and analyzed using a fluorescence microscopy system (Zeiss, Germany). At least 10 fields were counted for each slide. The ratio of TUNEL-positive cells was determined by calculating TUNEL-positive cells to the DAPI-positive cells in each field.

#### Phalloidin Staining

Phalloidin staining was performed according to the manufacturer’s instructions. In brief, the BV2 microglia were fixed in 4% PFA, washed by PBS three times, then incubated at room temperature with 0.5% Triton X-100 buffer for 5 min and washed again in PBS. FITC-phalloidin (1:200 diluted in 3% BSA, Yeasen, China) was added to the cover slips and incubated at room temperature in the dark for 30 min (Szabo et al., 2016). After washing three times with PBS, the nuclei of the cells were counterstained with DAPI (Beyotime, China). The FITC-labeled phalloidin was analyzed under a confocal microscope (Zeiss, Germany), and cell areas were measured using the ImageJ Software.

#### Real-Time Quantitative Polymerase Chain Reaction

RT quantitative PCR (RT-qPCR) was performed as previously described (Li Z. et al., 2016). Total RNA was extracted from the cell pellets using Trizol (Invitrogen, Carlsbad, CA, USA) according to the manufacturer’s instructions, and this step was followed by reverse transcription to determine the mRNA expression levels of IL-1β, TNF-α, IL-6, found in inflammatory zone 1 (Fizz1), chitinase-like 3 (Chil3/Ym1) and arginase 1 (Arg-1). Quantitative PCR amplification was performed in triplicate using the SYBR Green kit (Takara Company, Japan) with the following program: 1 cycle of 95°C for 30 s, 40 cycles of 95°C for 5 s and 60°C for 30 s. The primers used for this experiment are shown in Table 1. GAPDH served as the endogenous control gene to normalize the amplified signals of the target genes. The dissociation stages, melting curves and quantitative analyses of the data were performed using the Thermal Cycler Dice Real Time system (Takara Company).

**Table 1:** Primers used in Real-time quantitative polymerase chain reaction (RT-qPCR).

Genes	Forward	Reverse
TNF-α	5’-ACGTGGAACTGGCAGAAGAG -3’	5’-GGTCTGGGCCATAGAACTGA-3’
IL-1β	5’-GGCAACTGTTCCTGAACTCAACTG-3’	5’-CCATTGAGGTGGAGAGCTTTCAGC-3’
IL-6	5’-CTGGGAAATCGTGGAAATGAG -3’	5’-TCCAGTTTGGTAGCATCCATCA -3’
Fizz1	5’-TGTACTGCCATGGAGCTGAG-3’	5’-CAGGAAGAGCTGGCATAAGG-3’
Ym1	5’-GCCCACCAGGAAAGTACACA-3’	5’-CACGGCACCTCCTAAATTGT-3’
Arg-1	5’-CAGAACCTGCTGTCCTGTGA-3’	5’-TGTCGTTGGAATCAACCTGA-3’
GAPDH	5’-AGGTCGGTGTGAACGGATTTG-3’	5’-TGTAGACCATGTAGTTGAGGTCA-3’

#### Statistical Analysis

The data were analyzed by a one-way analysis of variance (ANOVA) followed by LSD *post hoc* analysis for multiple comparisons using SPSS 18.0 software (SPSS, Chicago, IL, USA). Data were expressed as the mean ± SEM, and *P* < 0.05 was considered statistically significant.

## Results

### EpoB Treatment Improved Behavioral Deficits in 6-OHDA-Lesioned Mice

**Figure 2:**
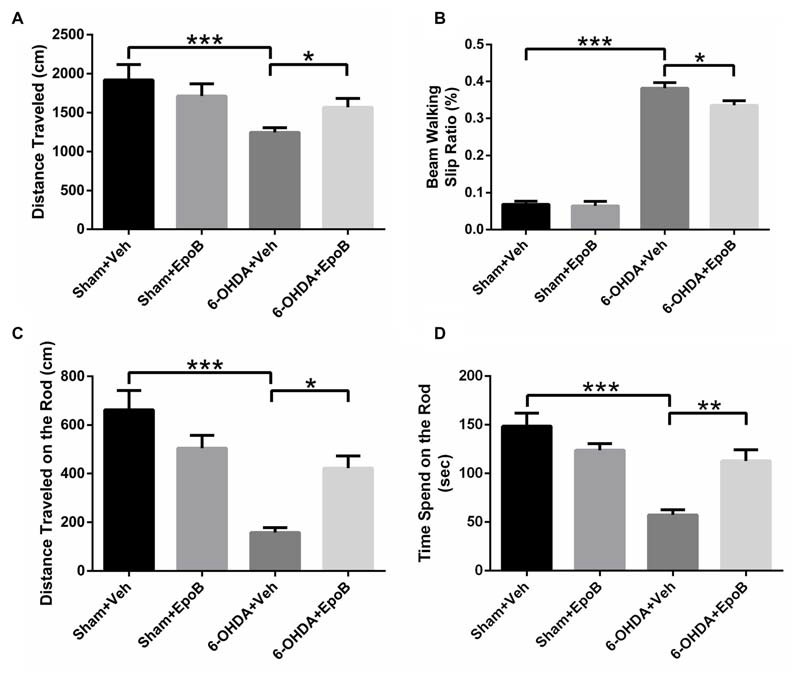
EpoB treatment improved behavioral deficits in 6-OHDA-lesioned mice. **(A)** Quantitative data of the total distance traveled in the open field test over a period of 5 min for each group. **(B)** Quantitative data of the incidence of foot slips in the beam walking test for each group. **(C,D)** Quantitative data of the distance traveled within 3 min **(C)** and time spent on the rod at 35 rpm **(D)** in the rotarod test for each group. Data are expressed as the means ± SEM (*n* = 10 for the behavior tests in each group; *P < 0.05, **P < 0.01 and ***P < 0.001).

To explore the effects of EpoB on 6-OHDA-induced motor impairment, we assessed the behavioral performance of the mice using the open field, balance beam walking and rotarod tests at 7 days after surgery. The open field test showed that the total distance traveled in the 6-OHDA-injected group was markedly decreased compared to that in the sham group (*F*_(3,36)_ = 17.20, *P* < 0.001, Figure 2A), whereas EpoB treatment significantly ameliorated the reduced movement of the 6-OHDA-lesioned mice (*F*_(3,36)_ = 17.20, *P* < 0.05, Figure 2A). In the beam walking test, mice in the sham group showed a good walking strategy that was performed by employing all four limbs. However, a significantly increased incidence of foot slips was observed in the 6-OHDA-lesioned mice compared with control mice (*F*_(3,36)_ = 193.86, *P* < 0.001, Figure 2B). EpoB treatment led to a significant recovery in steps on the beam walking performance test (*F*_(3,36)_ = 193.86, *P* < 0.05, Figure 2B). A rotarod test was carried out to assess coordination capability of the mice. Compared to the control group, the distance traveled within 3 min and time of stay on the rod at 35 rpm significantly decreased in 6-OHDA-treated mice, while EpoB treatment caused a significant increase in the distance traveled (*F*_(3,36)_ = 19.86, *P* < 0.05, Figure 2C) and time of keeping on the rod (*F*_(3,36)_ = 15.40, *P* < 0.01, Figure 2D). Compared with the control group, EpoB alone did not induce significant differences in the total distance in the open field, incidence of faulty steps in the beam walking performance test or distance traveled and time of staying on the rod in normal mice, indicating that the EpoB had no effect on the motor performance in control mice.

### EpoB Protected the Nigrostriatal Dopaminergic Projection Against 6-OHDA-Induced Neurotoxicity

**Figure 3:**
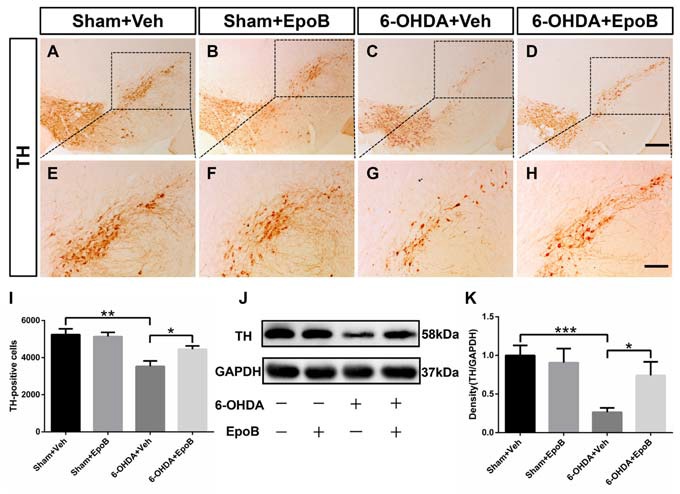
EpoB administration protected TH-positive (TH^+^) neurons against 6-OHDA neurotoxicity in the substantia nigra. **(A–H)** Representative immunohistochemistry staining of TH in the substantia nigra compacta (SNc) area for each group 7 days after 6-OHDA injection and EpoB treatment. **(I)** Quantitative data of TH^+^ neurons in the SNc for each group 7 days after 6-OHDA injection and EpoB treatment. **(J–K)** Representative western blot image and quantitative data of TH protein expression in the SN for each group 7 days after 6-OHDA injection and EpoB treatment. Data are expressed as the means ± SEM (*n* = 5 for staining of TH in each group; *n* = 3 for western blot of TH in each group; *P < 0.05, **P < 0.01 and ***P < 0.001). Scale bar in **(D)** = 200 μm and applies to **(A–D)** in **(H)** = 100 μm and applies to **(E–H)**.

**Figure 4:**
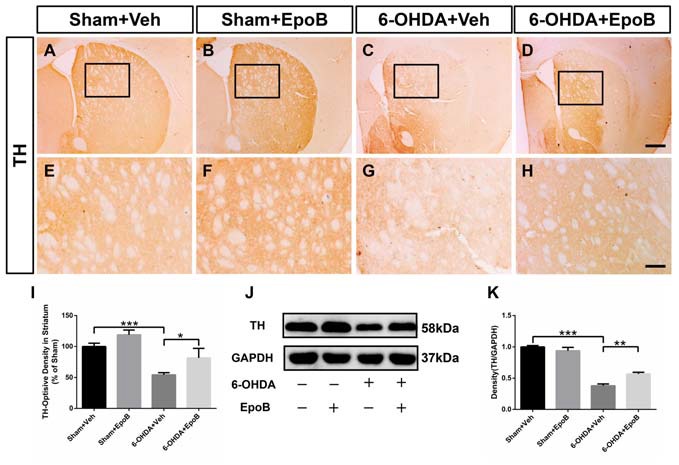
EpoB administration protected TH^+^ fibers in the striatum. **(A–H)** Representative immunohistochemistry staining of TH in the striatum for each group 7 days after 6-OHDA injection and EpoB treatment. **(I)** Quantitative data of the optical density of TH^+^ fibers in the striatum for each group 7 days after 6-OHDA injection and EpoB treatment. **(J,K)** Representative western blot image and quantitative data of TH protein expression in the striatum for each group 7 days after 6-OHDA injection and EpoB treatment. Data are expressed as the means ± SEM (*n* = 5 for staining of TH in each group; *n* = 3 for western blot of TH in each group; *P < 0.05, **P < 0.01 and ***P < 0.001). Scale bar in **(D)** = 200 μm and applies to **(A–D)** in **(H)** = 100 μm and applies to **(E–H)**.

To determine the neuroprotective potential of EpoB on DA neuronal degeneration in the nigrostriatal pathway, we stained TH^+^ neurons and fibers in the SN (Figures 3A–H) and striatum (Figures 4A–H), respectively. One week after lesion, the histological analysis of the right-sided SN displayed a significant decrease (about 33%) in TH^+^ neurons in 6-OHDA-lesioned mice (Sham+Vehicle: 5240 ± 314.8 cells vs. 6-OHDA+Vehicle: 3520 ± 305.3 cells; *F*_(3,16)_ = 8.00, *P* < 0.01, Figures 3A,E,C,G,I) whereas EpoB administration significantly reduced the toxicity of 6-OHDA and markedly improved the number of TH^+^ neurons in the SN (*F*_(3,16)_ = 8.00, *P* < 0.001, 6-OHDA+Vehicle group vs. 6-OHDA+EpoB group, *P* < 0.05, Figures 3C,D,G,H,I). Western blot analysis confirmed that TH expression in the SN markedly decreased in the 6-OHDA-lesioned group, while TH expression significantly improved in the EpoB-treated 6-OHDA-lesioned group (Figures 3J,K).

To determine the protective effect of EpoB on the nigrostriatal fibers after 6-OHDA lesion, the density of TH^+^ fibers in the striatum was investigated. It was markedly reduced in 6-OHDA-injured mice (Sham+Vehicle: 100.0 ± 5.4% vs. 6-OHDA+Vehicle: 54.4 ± 3.5%, *F*_(3,16)_ = 9.07, *P* < 0.001, Figures 4A,E,C,G,I). EpoB treatment significantly ameliorated the TH^+^ fiber loss in the striatum (*F*_(3,16)_ = 9.07, *P* < 0.001, 6-OHDA+Vehicle group vs. 6-OHDA+EpoB group, *P* < 0.05, Figures 4C,D,G,H,I). Western blot analysis confirmed that the TH level in the striatum was significantly decreased in the 6-OHDA-lesioned group, while EpoB treatment rescued the decline in TH level (Figures 4J,K).

### Effects of EpoB on the Striatal Levels of DA and Its Metabolites in the 6-OHDA-Injured Mice

**Figure 5:**
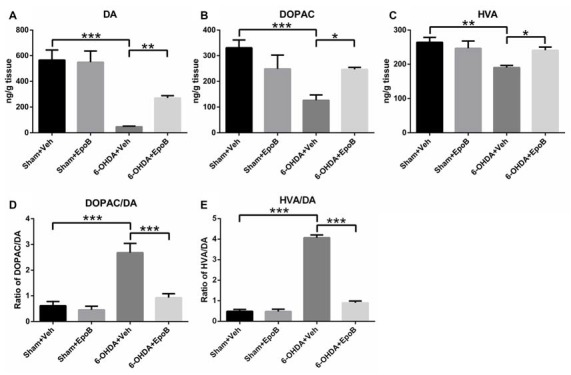
The effects of EpoB on the levels of DA and its metabolites 3,4-dihydroxyphenylace-tic acid (DOPAC) and homovanilic acid (HVA) in the striatum. **(A–C)** Quantitative data of DA, DOPAC and HVA contents in the striatum for each group 7 days after 6-OHDA injection and EpoB treatment. **(D,E)** The ratio of DOPAC/DA and HVA/DA in the striatum for each group 7 days after 6-OHDA injection and EpoB treatment. Data are expressed as the means ± SEM (*n* = 4 for each group; *P < 0.05, **P < 0.01 and ***P < 0.001).

HPLC analysis of DA and its metabolites in the striatum of mice was performed to correlate the histological results of TH-fiber density. As shown in Figure 5, the concentrations of DA, DOPAC and HVA in the lesioned striatum of 6-OHDA+Veh group were markedly reduced to 8.2%, 38.1%, and 71.9%, respectively, as compared to concentrations in the sham group. Treatment with EpoB increased the striatal concentrations of DA, DOPAC and HVA to about 5.8-fold, 1.95-fold and 1.3-fold, respectively, as compared to the 6-OHDA-lesioned group.

In addition, the DOPAC/DA ratio, indicating the monoamine oxidase (MAO)-dependent DA catabolism, was markedly increased in the 6-OHDA-injured mice, and EpoB treatment strongly suppressed the 6-OHDA-induced MAO-dependent DA catabolism and significantly decreased the DOPAC/DA ratio (*F*_(3,12)_ = 79.94, *P* < 0.001, Figure 5D). The rate of the total DA catabolism was expressed as the HVA/DA ratio, and this ratio was significantly increased in the striatum of 6-OHDA-injured mice (*F*_(3,12)_ = 80.23, *P* < 0.001, Figure 5E), while EpoB treatment significantly decreased the HVA/DA ratio of 6-OHDA-injured mice (*F*_(3,12)_ = 80.23, *P* < 0.001, Figure 5E). These results demonstrated that EpoB protected against 6-OHDA-induced neurotransmitter deficiency both through protection of the DA neurons and through modulation of DA catabolism.

### EpoB Attenuated Microglia Activation Induced by 6-OHDA in the SNc

**Figure 6:**
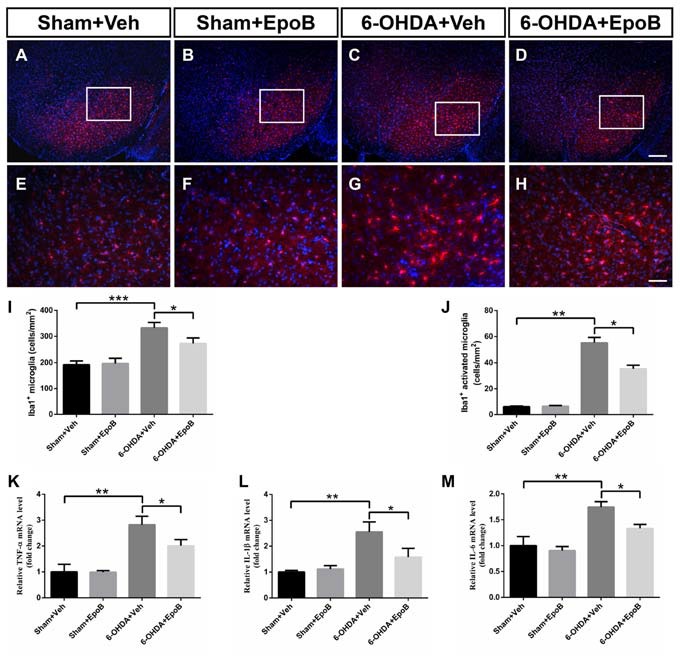
EpoB administration alleviated microglial activation in the SNc 7 days after 6-OHDA-injuried. **(A–H)** Representative immunofluorescence staining of Iba1^+^ cells in the SNc 7 days after 6-OHDA injection and EpoB treatment. **(I,J)** Quantitative data of Iba1^+^ total microglia **(I)** and Iba1^+^ activated microglia **(J)** in the SNc 7 days after 6-OHDA injection and EpoB treatment. **(K–M)** Quantitative data of tumor necrosis factor (TNF)-α **(K)** interleukin (IL)-1β **(L)** and IL-6 **(M)** expression in the SNc 7 days after 6-OHDA injection and EpoB treatment. Data are expressed as the means ± SEM (*n* = 5 for staining of Iba1 in each group; *n* = 3 for RT-PCR in each group; *P < 0.05, **P < 0.01 and ***P < 0.001). Scale bar in **(G)** = 200 μm and applies to **(A–D)** in **(H)** = 50 μm and applies to **(E–H)**.

We performed immunofluorescence to assess microglia activation in the SNc at Day 7 after 6-OHDA injury (Figures 6A–H). It showed a significantly increased total number of microglia in the SNc (*F*_(3,16)_ = 12.8, *P* < 0.001, 6-OHDA+Vehicle group vs. Sham+Vehicle group, *P* < 0.001, Figures 6A,C,E,G,I) whereas EpoB treatment dramatically reduced the number of microglia in the SNc (*F*_(3,16)_ = 12.8, *P* < 0.001, 6-OHDA+Vehicle group vs. 6-OHDA+EpoB group, *P* < 0.05, Figures 6C,D,G,H,I). We found that activated Iba1^+^ labeled microglia were increased markedly by 6-OHDA treatment, which could be suppressed by EpoB treatment (Figure 6J). Treatment with EpoB did not influence the total number of Iba1^+^ and activated Iba1^+^ labeled microglia in the SNc of the control mice (Figures 6B,F). Further experiments showed that the levels of TNF-α, IL-1β and IL-6 in the SN were markedly increased in the brain following 6-OHDA injury and that EpoB treatment significantly reduced their expression (Figures 6K–M). These results suggested that EpoB might exert beneficial effects by inhibiting microglia activation induced by 6-OHDA neurotoxicity.

### EpoB Reduced the Cytotoxicity on Dopaminergic Cells Induced by 6-OHDA

**Figure 7:**
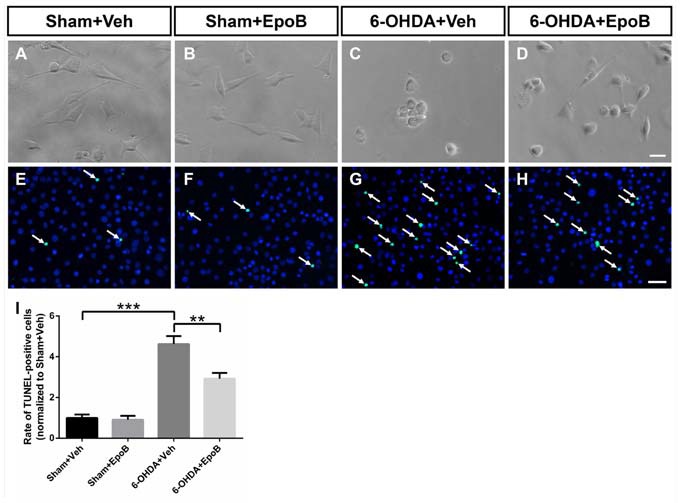
EpoB reduced the cytotoxicity on MN9D cells co-cultured with BV2 cells induced by 6-OHDA stimulation. **(A–D)** Representative morphological photographs of MN9D cells co-cultured with BV2 cells after 6-OHDA and EpoB treatment. **(E–H)** Representative immunofluorescence staining images of DAPI (blue) and TUNEL-positive (green) of MN9D cells co-cultured with BV2 cells after 6-OHDA and EpoB treatment. **(I)** Statistics for rate of TUNEL-positive MN9D cells. Data are expressed as the means ± SEM (*n* = 5 for each group; **P < 0.01 and ***P < 0.001). Scale bar in **(D)** = 30 μm and applies to **(A–D)** in **(H)** = 50 μm and applies to **(E–H)**.

We further investigated the neuroprotective effect of EpoB against 6-OHDA stimulation using a BV2 microglia/MN9D DA neurons transwell co-culture system. As shown in Figures 7A–D, phase contrast microscopy showed that 6-OHDA stimulation drastically decreased the number of adhering cells and destroyed the neurites in MN9D cells. Treatments with EpoB were able to substantially rescue the neuritic dystrophy in MN9D cells. We also analyzed the apoptosis of MN9D cells with TUNEL staining and demonstrated that 6-OHDA treatment increased the ratio of TUNEL-positive MN9D cells about four-fold, while treatment with EpoB for 12 h rescued MN9D cells from apoptosis (Figures 7E–I). It suggested that the application of EpoB to microglia protected the MN9D cells from cytotoxicity.

### EpoB Redistributed the Cytoskeleton and Caused the Morphological Transition of the Microglia

**Figure 8:**
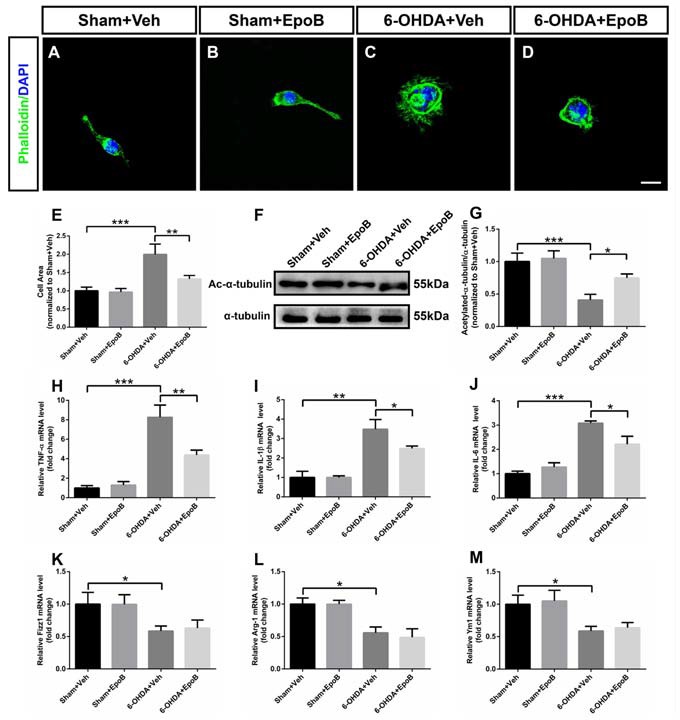
Effects of EpoB and 6-OHDA on morphological transition and inflammatory factors in the processing of BV2 microglia polarization to the M1 or M2 phenotype when co-cultured with MN9D cells. **(A–D)** BV2 microglia were stained with phalloidin. **(E)** Quantitative data of cell areas of BV2 microglia treated with 6-OHDA and/or EpoB. **(F)** Effect of EpoB on the level of acetylated microtubules of BV2 microglia co-cultured with MN9D cells after 6-OHDA and EpoB treatment. **(G)** Quantitative data of acetylated microtubules. **(H–J)** Quantitative data of relative mRNA levels for TNF-α **(H)** IL-1β **(I)** and IL-6 **(J)** for M1 makers. **(K–M)** Quantitative data of relative mRNA levels for Fizz1 **(K)** Arg-1 **(L)** and Ym1 **(M)** for M2 makers. Data are expressed as the means ± SEM (*n* = 5 for phalloidin staining in each group; *n* = 3 for RT-PCR in each group; *P < 0.05, **P < 0.01 and ***P < 0.001). Scale bar in **(H)** = 30 μm and applies to **(A–H)**.

It has been indicated that neuronal apoptosis induces a microglia phenotypic switch from a homeostatic to neurodegenerative phenotype (Keren-Shaul et al., 2017; Krasemann et al., 2017). As the morphological transition of microglia cells closely related with their functions, we explored the shape transformation of BV2 microglia co-cultured with MN9D neurons when treated with 6-OHDA and/or EpoB. In phalloidin staining, BV2 microglia displayed a distinctive unipolar shape characterized with one or more processes in the Sham+Veh or Sham+EpoB group (Figures 8A,B). After 6-OHDA treatment, the BV2 cells rapidly transformed into flattened shape characterized with increased surface area (Figures 8C,E). However, microglia co-cultured with MN9D neurons treated with EpoB after 6-OHDA stimulation showed preserved morphology with a smaller surface area resembling closely to unstimulated microglia in the control plates (Figures 8D,E). The morphological transition of microglia cells was usually caused by cytoskeletal elements such as microtubules. In the present study, it demonstrated that the level of acetylated microtubules in the BV2 microglia co-cultured with MN9D neurons were markedly reduced by 6-OHDA stimulation (Figures 8F,G), which could be antagonized by EpoB treatment (Figures 8F,G). Combining the phalloidin staining data and the western blot data, this indicated that the transition of microglia to a ramified phenotype was accompanied by increased microtubule modification.

### EpoB Regulated the BV2 Microglia M1/M2 Polarization in the Co-culture System of MN9D/BV2 Cells After 6-OHDA Treatment

To evaluate M1/M2 polarization, we analyzed the expression of M1 (TNF-α, IL-1β and IL-6) and M2 polarization (Fizz1, Ym1 and Arg-1) markers, respectively, in BV2 cells co-cultured with MN9D cells using RT-PCR. It indicated that treatment with 6-OHDA for 24 h promoted microglial M1 polarization by dramatically increasing mRNA levels of TNF-α, IL-1β and IL-6 (Figures 8H–J). Interestingly, the application of EpoB for 12 h followed by 6-OHDA administration significantly blocked the increase in TNF-α, IL-1β and IL-6 expression in BV2 microglia cells (Figures 8H–J). Meanwhile, we also noticed that 6-OHDA inhibited M2 polarization of BV2 cells by dramatically decreasing the mRNA levels of Fizz1, Arg-1 and Ym1 (Figures 8K–M). However, the application of EpoB for 12 h after 6-OHDA administration did not influence the mRNA levels of Fizz1, Arg-1 and Ym1 (Figures 8K–M). Collectively, our findings indicated that EpoB might abolish 6-OHDA-induced microglial M1 polarization by inhibiting the expression of pro-inflammatory factors without affecting the expression of anti-inflammatory factors, such as Arg-1, Ym1 and Fizz1 when co-cultured with MN9D cells.

## Discussion

In the present study, we demonstrated that EpoB treatment attenuated the behavioral abnormalities induced by 6-OHDA injury in mice. It was revealed that the behavioral improvement of EpoB was closely related to the protection of nigrostriatal DA. Our data suggested that EpoB redistributed the cytoskeletal elements and caused the transition of the microglia from flattened shape to a ramified phenotype, and inhibited the expression of pro-inflammatory cytokines produced by M1 phenotype microglia, which was critical for the neuroprotection of EpoB against 6-OHDA injury.

We found that 6-OHDA treatment induced behavioral deficits in mice, such as a reduction in spontaneous motor behavior and motor incoordination, and these behavioral alterations were consistent with previous reports (Su et al., 2018). Behavioral impairments observed in the 6-OHDA-induced animal model of PD correlated with nigrostriatal neurodegeneration and depletion of DA and metabolites in the striatum. Of particular importance, EpoB treatment prevented the reduction in TH^+^ cells and the loss of DA and metabolites in the striatum. Our results suggested that EpoB can attenuate the decrease in the levels of DA, DOPAC and HVA in the striatum of 6-OHDA-lesioned mice. Moreover, the present study showed that EpoB strongly depressed the 6-OHDA-evoked acceleration of MAO-dependent DA catabolism, indicated by the decreased DOPAC/DA ratio. Furthermore, a decrease in DA turnover induced by EpoB administration, demonstrated by the HVA/DA ratio, was also observed in this study.

EpoB is an FDA-approved, antineoplastic drug that has been used clinically in the treatment of ovarian cancer (Griffin et al., 2003). Instead of inducing apoptosis in tumor cells at therapeutic doses, subtoxic concentrations of EpoB have been shown to promote regeneration of axons and suppress formation of the glia scar in spinal cord injury models (Ruschel et al., 2015). Besides its microtubule stability function, EpoB was confirmed to have an immunoregulation function such as increasing the secretion of macrophage colony-stimulating factor (M-CSF) during regeneration in spinal cord injury (Mao et al., 2017). Inflammation has been recognized as playing an important role in the pathogenesis of PD (Kannarkat et al., 2013). Several reports showed that activated microglia and increases in the levels of pro-inflammatory cytokines observed in the brains of PD patients may further exacerbate neurodegeneration (Moehle and West, 2015; Ferreira and Romero-Ramos, 2018). Moreover, there are many reports showing that excessive microglial activation can cause neurotoxicity in the nigrostriatal DA system *in vivo* (Mullin and Schapira, 2015; Johnson et al., 2018). This is consistent with our present results in which we demonstrated that a subtoxic dosage of EpoB inhibited the increase in the total number of microglia and activated microglia in the SNc of 6-OHDA-injured mice. It was further confirmed in the present study that EpoB produced anti-inflammation by regulating the release of pro-inflammatory cytokines, including TNF-α, IL-1β and IL-6, in the SN of the 6-OHDA-injured mouse model.

In line with the *in vivo* finding, using a transwell co-culture system, we discovered that EpoB could protect MN9D neurons co-cultured with BV2 cells against from cytotoxicity induced by 6-OHDA stimulation. Recent studies have suggested the dying/dead DA neurons release neurotoxic soluble factors such as *α*-synuclein and damage-associated molecular patterns (DAMPs), which in turn drive and maintain reactive microgliosis in neurodegenerative diseases (da Silva et al., 2016; Freeman et al., 2017). The present study further demonstrated that mRNA levels of the M1-type of pro-inflammatory cytokines including TNF-α, IL-1β and IL-6 were markedly up-regulated, while M2-type cytokines including Fizz1, Ym1 and Arg-1 were significantly inhibited in BV2 cells after 6-OHDA treatment. EpoB treatment inhibited the increase in the levels of TNF-α, IL-1β and IL-6 in 6-OHDA-stimulated BV2 cells co-cultured with MN9D neurons, while EpoB treatment did not influence the level of Fizz1, Ym1 and Arg-1. These results suggested that EpoB can inhibit the 6-OHDA-induced microglial M1 state. Our recent study demonstrated that the neuroprotection of EpoB resulted from an increased acetylated α-tubulin level and maintenance of microtubule stabilization of DA neurons after intracerebral hemorrhage (Yang et al., 2018a,b). During the morphological transition of microglia from an activated ameboid phenotype to a less-active ramified phenotype, the changes in microtubule composition and reorganization of the microtubule skeleton play key roles and form a more stable microtubule network (Ilschner and Brandt, 1996). In the present study, phalloidin staining and analysis of acetylated α-tubulin level indicated that EpoB might directly act on the microtubule skeleton of microglia and enhance the morphological transition from an ameboid phenotype to a ramified phenotype.

The roles of microglia function in neurodegenerative diseases is complicated due to its heterogeneity. Noticeably, recent study has described a novel microglia type associated with neurodegenerative diseases (DAM), which shows strong phagocytic ability and has the potential to restrict neurodegeneration (Keren-Shaul et al., 2017). Dysregulation of homeostatic microglia might be involved in the pathogenesis of neurodegenerative disorders. Whether EpoB could modify the microtubule skeleton of microglia to exert their phagocytic function in PD is need to be further studied.

In summary, we provided the first preclinical evidence of EpoB-mediated neuroprotection in this model of PD. Additionally, we inferred the effect of EpoB on microglial activation and polarization after 6-OHDA administration. These findings indicated that EpoB-induced modulation of the immune response is an important neurobiological mechanism against 6-OHDA-induced behavioral impairment in this PD model. As the subtoxic dosages of EpoB presented no obvious adverse side effects (Ruschel et al., 2015), EpoB might be used clinically to delay the progression of neurodegenerative diseases.

## Author Contributions

ZY, LY and YY conducted the experiments, collected and analyzed the data and drafted the manuscript. ZY, DS and SC contributed to acquisition and analysis of data. ZY, HX and XF designed the experiments, supervised the project and revised the manuscript.

## Conflict of Interest Statement

The authors declare that the research was conducted in the absence of any commercial or financial relationships that could be construed as a potential conflict of interest. The reviewer ACJ and handling Editor declared their shared affiliation at the time of the review.
